# Accuracy of swept source biometry device in detecting macular diseases compared with swept source OCT

**DOI:** 10.1186/s13104-023-06641-3

**Published:** 2023-12-19

**Authors:** Sidra Zafar, Taha Muneer Ahmed, Rashid Baig, Irfan Jeeva, M. A. Rehman Siddiqui

**Affiliations:** 1 Department of Ophthalmology and Visual Sciences, Aga Khan University Hospital, Karachi, Pakistan; 2 Shahzad Eye Hospital, Shahzad Eye Hospital, Gulshan-e-Iqbal, A-16, Karachi, Pakistan; 3 The Eye Centre, South City Hospital, Karachi, Pakistan

**Keywords:** SS-OCT, Biometry, Cataract surgery, Premium IOLs, Refractive surprises, Patient satisfaction

## Abstract

**Objective:**

To assess the diagnostic accuracy of the IOLMaster 700 foveal scans to detect foveal pathology compared with a standard swept-source optical coherence tomography (SS-OCT) device**.**

**Results:**

One hundred seventy eye scans of 95 patients were included in the final analyses. Ninety-nine (58.2%) scans were classified as abnormal by SS-OCT. Mean sensitivity of the biometry device was 67.5% (range: 51–84%) and mean specificity was 69.5% (range: 44–95%). Intra-class correlation coefficients were 0.912 and 0.835, for reader 1 and 2, respectively. Area under the curve for receiver operating curve was 0.726. Foveal scans of the IOLMaster 700 can provide clinically useful information. Clinicians should pay attention to the macular scans when reviewing biometry prior to cataract surgery and standard macular OCT should ideally be supplemented in suspicious cases.

## Introduction

Occult macular pathology may be missed on routine pre-operative fundoscopic examination, particularly in the setting of media opacities such as visually significant cataract. This can negatively affect post-operative visual outcomes, and patient satisfaction following an otherwise successful phacoemulsification procedure. Furthermore, in some cases this may also lead to litigation, and/ or additional surgical procedures. Recently, there have been suggestions that optical coherence tomography (OCT) should be used to screen macular pathology in patients undergoing cataract surgery [1, 2]. OCT is extensively used in ophthalmology clinics today for the assessment, and management of retinal diseases. Swept source OCT (SS-OCT) can better evaluate posterior segment disease, even in the presence of cataract due to its unique laser wavelength and better penetrance [3, 4].

Zeiss IOLMaster 700 (Carl Zeiss Meditec AG, Germany) utilizes swept source OCT technology for highly precise biometric measurements. In addition, a foveal scan is also generated. The foveal scan of the biometry device was introduced as a quality control of patient's fixation during biometry [5, 6]. However, this scan can also provide valuable information on macular pathology and may help avoid post-operative surprises.

The aim of our study was to assess the sensitivity and specificity of the foveal scan of the IOLMaster 700 to detect macular disease, compared with a standard SS-OCT device.

## Patient and methods

This was a retrospective study carried out at the Shahzad Eye Hospital, Karachi, Pakistan. The study complied with the declaration of Helsinki and was conducted after exemption from the Shahzad Eye Hospital ethical review committee. Successive patients who underwent scans with IOLMaster 700 (Carl Zeiss Meditec AG, Germany) and imaging on an SS-OCT machine (Triton; Topcon Inc., Tokyo, Japan) over a 6 month period were included.

IOL Master 700 is a noncontact optical biometry device that combines keratometric measurement with swept-source OCT scanning. Foveal scans are automatically acquired. Imaging was performed by a single trained technician under standardized conditions. Foveal scans obtained from the biometer were read independently by two non-vitreoretinal comprehensive ophthalmologists. Examiners were asked to assess if the scans were normal or abnormal. If the scans were abnormal, an attempt was made to identify the pathology on the foveal scan by the biometry device. Each scan was evaluated twice to assess intra-reader reliability.

SS-OCT images were read by a single fellowship trained vitreoretinal specialist. The cross-hair imaging protocol was used to evaluate for the presence of pathology. This imaging protocol generates two 6 mm lines centred on fovea. For every patient, scans of both eyes were evaluated (when available) and only pathology involving the fovea was considered.

## Results

Scans from 184 eyes of 102 eligible patients were identified during the study period. SS-OCT scans of 6 eyes could not be evaluated and were excluded. Similarly, 8 foveal scans from the biometry device were deemed unreadable by both readers. These eyes were also excluded. The remaining 170 eyes of 95 patients were included in the study.

**Fig. 1:**
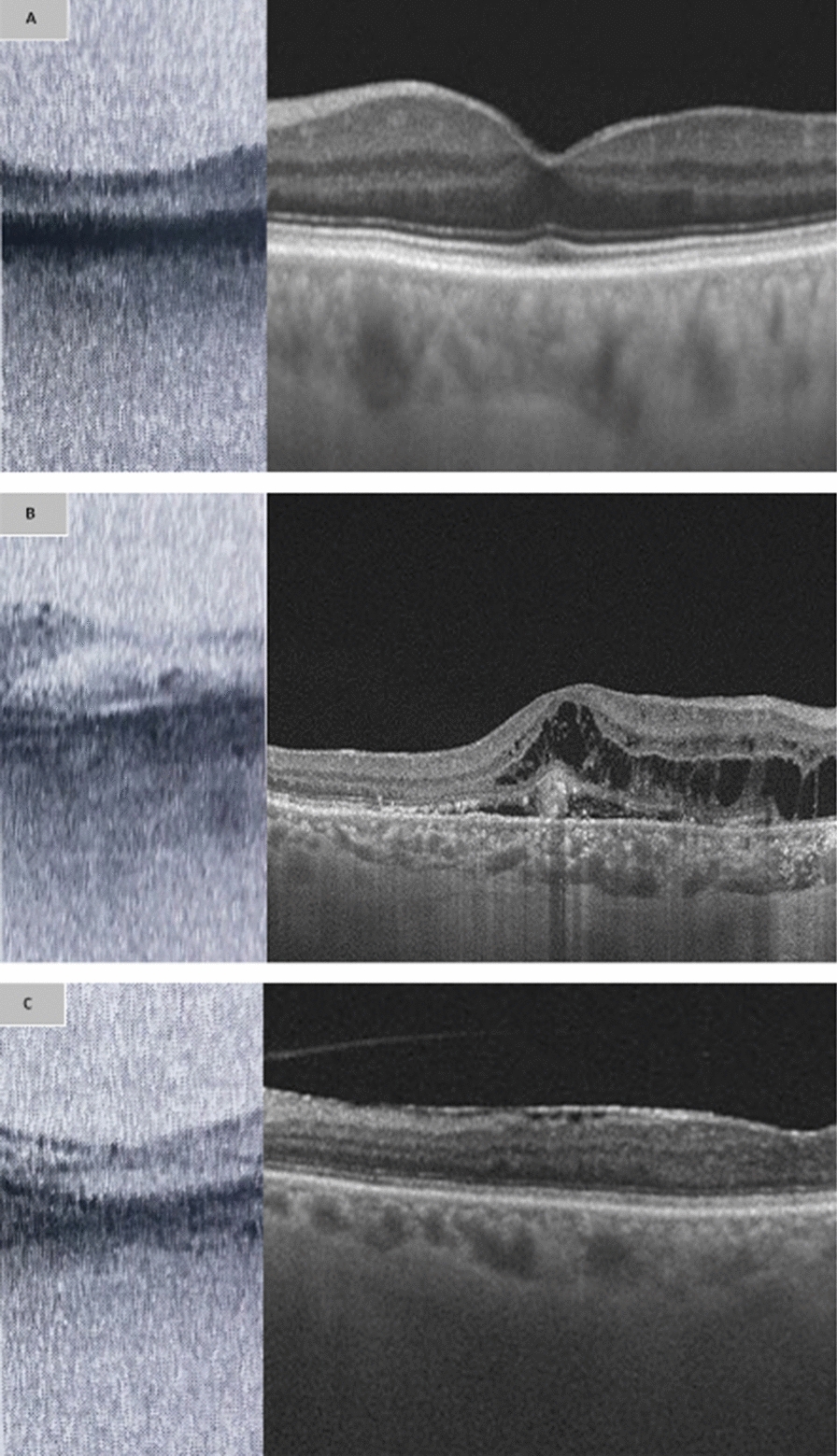
One mm foveal scans of the IOLMaster 700 compared with swept source OCT (SS-OCT) scans. **A** depicts a normal foveal scan on both biometry device and SS-OCT. **B** depicts intra-retinal edema with CNVM and **C** shows a scan of epiretinal membrane (ERM) on scans of both devices

Mean age of the included participants was 63 years ± 9.0 and 52% were males. Pathology was identified on 99 (58.2%) SS-OCT scans and 26 eyes were affected by more than one disease process. Edema including cystoid macular edema, intra-retinal and sub-retinal fluid was the most common abnormality, observed in 44 eyes (32.4%). This was followed by epiretinal membrane (ERM), which was identified in 23 eye scans (17%). A comparison of macular scans from both the biometry device and standard SS-OCT can be observed in Fig. 1A, B, C.

Pathological SS-OCT scans were further grouped into 2 main categories; A) scans with visually significant pathology and B) scans with visually insignificant pathology. Of the 99 abnormal scans, 22 scans were categorised as group B (22.2%). Changes were minor (mild ERM, n = 5, drusen, n = 6 and exudates, n = 4) or the pathologies were outside the foveal centre (n = 7). Evaluation of foveal scans for group B patients varied between both readers. With the exception of 1 scan, reader 1 categorized the remaining 21 scans as normal. Reader 2 however, categorized only 10 scans as normal.

**Fig. 2:**
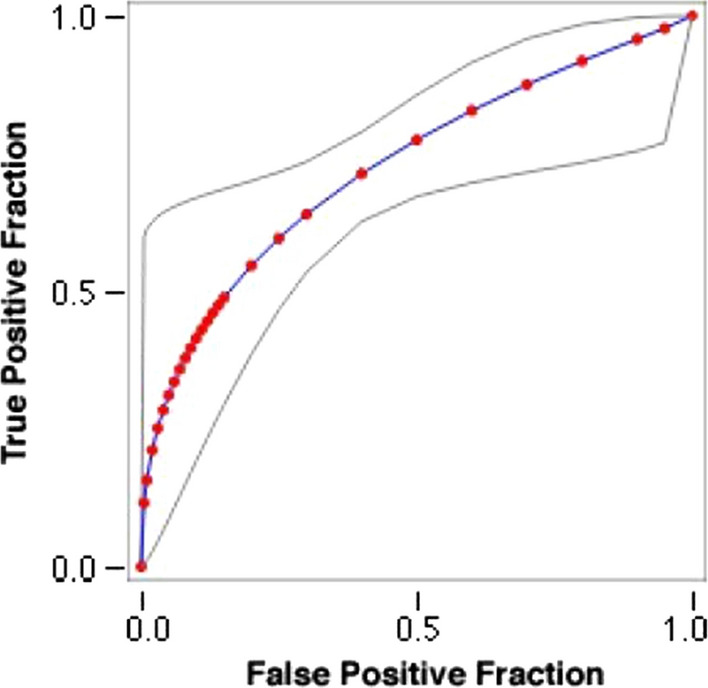
The receiver operating curve (ROC) of the IOLMaster 700, for detection of macular pathology in patients undergoing cataract surgery. The curve is depicted by the continuous line as a function of sensitivity plotted against 1-specificity. Area under the curve (AUC) is 0.726

For the biometric device’s foveal scans, 46/170 and 52/170 were categorized as normal and abnormal respectively, by both readers. Intra-reader reliability coefficient (ICC) for reader 1 was 0.912 (95% confidence interval; 0.870–0.941). ICC for the 2nd reader was 0.835 (95% confidence interval; 0.745–0.888). The receiver operating curve ROC curve is shown in Fig. 2. The area under the curve (AUC) was 0.726.

Mean sensitivity of the IOLMaster foveal scans at disease detection was 67.5% (range: 51–84%). Mean specificity was 69.5% (range: 44–95%). Compared to standard SS-OCT scans, diagnostic accuracy of the biometry device scans was 68.3% (range: 66.5–70%).

## Discussion

In recent years, a growing body of evidence recognising the value of preoperative OCT screening for cataract surgery has emerged [7]. This work stems from the fact that it is crucial to have an understanding of the health of macula before conducting cataract surgery because this information can determine patient’s visual outcome, identify potential issues in selecting intraocular lenses, and influence postoperative expectations [7]. The routine fundus examination, based on cataract characteristics during presentation, might not be able to detect subtle macular pathologies effectively [8]. This limitation can be attributed to factors such as a dense cataract obstructing the view of the fundus, insufficient pupillary dilation, or photophobia experienced by the patient [7]. Consequently, important pathologies in the posterior segment of the eye may remain undetected, impacting the patient's final visual outcome after surgery [7]. Review of current literature on this indicates that preoperative OCT results in an occult maculopathy detection rate ranging from 4.6 to 54.2% [7–9]. The types of macular pathology most frequently detected through this process are interface abnormalities followed by macular degeneration [7]. Other commonly uncovered pathology is macular oedema and full thickness macular holes [7]. Postoperative discovery of such pathology may lead to patient dissatisfaction, particularly among those who choose premium intraocular lenses (IOLs) [10]. Moreover, it may also result in legal action due to the failure to discuss pre-operative pathologies during the consent process [7]. Despite this OCT is not routinely employed during traditional preoperative workup due to concerns revolving around cost, availability and clinical work load [7]. The IOLMaster 700, however, brings SS-OCT capabilities to biometry and could potentially address all three of these major obstacles to more widespread preoperative OCT screening.

Other studies have compared the diagnostic accuracy of currently available SS-OCT devices as done in our study [6, 11–13]. These studies have similarly evaluated the diagnostic utility of biometric SS-OCT and have reported a sensitivity ranging from 46.0 to 81.0%. There are a number of reasons for the wide range of sensitivity reported for this modality. Firstly, the biometer has a limited coverage area (central 1-mm of the fovea) in the scan which omits the detection of any macular abnormalities beyond this region. In one prior study, eyes that appeared normal on SS-OCT biometry device were erroneously categorized as normal because their corresponding SD-OCT scan displayed extrafoveal abnormalities that could not be detected on SS-OCT biometer [12]. Additionally, the IOLMaster 700's SS-OCT has a low axial resolution which could contribute to the low sensitivity. Despite this subpar sensitivity of SS-OCT biometry however, the specificity of the IOLMaster in all included studies was promising (83.2%-93.0%). This suggests that while the IOLMaster may not be an effective screening tool, an abnormal IOLMaster scan is a strong indicator of an occult pathology and should prompt further evaluation[6, 12].

In our study, the diagnostic accuracy of the IOLMaster (68.3%) was comparable with prior studies (6, 11, 12). Because cataract surgeries are mostly performed by comprehensive ophthalmologists, we believed it would be more generalizable if comprehensive ophthalmologists read the biometry scans and only employed comprehensive ophthalmologists to interpret the obtained biometry scans as opposed to vitreoretinal specialists. We believe this expands the generalizability of our study.

Notably, our study reports moderate specificity (69.5%) of the IOLMaster compared to prior reports in the literature (83.2–93.0%). While this may be due, in part, to the patients in our cohort being recruited from a vitreoretinal clinic leading to the readers having a tendency to overcall pathology, this suggests that nearly 1 in 3 patients diagnosed with disease on the IOLMaster scans may not have disease when subsequently evaluated on traditional OCT. This is clinically relevant as false positives in this will invariably necessitate further unnecessary clinical workup which could disrupt the workflow of a busy surgical practice. This is especially relevant given that our study was the first among current similar studies to be conducted in a resource-limited developing country where unnecessary utilization of resources may be particularly taxing on patients and health systems. While the IOLMaster may have utility in screening the eyes for significant macular pathology, positive findings should be carefully evaluated on an individual basis as false positives may be more encountered than previously reported.

There are certain limitations in our study. First, this was a retrospective study and therefore limited by design. Future prospective studies investigating this topic should be conducted to corroborate results. Second, our study was limited by the diagnosing clinicians being aware of the study being undertaken as well as being aware that patients were recruited exclusively from a vitreoretinal clinic. This not only resulted in a greater proportion of pathology in our study compared too prior studies but may have biased judgements, introduced observer bias and led to overcalling of pathology by readers in our study. Lastly, the SS-OCT scans in our study were read by a single vitreoretinal surgeon and were not cross verified. This could have potentially imparted a diagnostic bias in the classification of cases.

## Data Availability

The datasets used and/or analysed during the current study are not publicly available due to institutional and national data privacy laws.
